# Impact of Metabolic Syndrome Diagnosis and Its Treatment on Survival of Colorectal Cancer Patients

**DOI:** 10.1155/2019/6527457

**Published:** 2019-04-21

**Authors:** Rose N. Mafiana, Maimona S. Al-Kindi, Ngozichukwu Mafiana, Ahmed S. Al Lawati, Mansour Al Moundhri

**Affiliations:** ^1^Department of Pharmacy, Sultan Qaboos University Hospital, Oman; ^2^Oman Medical College, Oman; ^3^Department of Internal Medicine, College of Medicine and Health Sciences, Sultan Qaboos University, Oman

## Abstract

**Background:**

Epidemiologic findings on the effect of metabolic syndrome (MetS) and its treatment on colorectal cancer (CRC) survival have been inconsistent and have not been previously studied in an Arab population such as the Omani population.

**Patients and Methods:**

Data from the hospital records of 301 CRC patients treated in Sultan Qaboos University (SQUH), Oman, from 2006 to 2014 were analyzed retrospectively to determine the effects of MetS and its treatment on CRC survival. Overall survival (OS) by MetS status and by medications for MetS components management was compared with Cox proportional models.

**Results:**

Of the 301 patients, 76 (25.2%) had MetS, 20.3% were on insulin, 23.9% were on metformin, 25.6% took statins, 17.9% were on either angiotensin converting enzyme inhibitors (ACEI) or angiotensin receptor blocker (ARB). Whereas metformin (HR, 0.46, 95% CI, 0.25-0.84) and statins (HR, 0.58; 95% CI, 0.35-0.96) had a protective effect on OS, insulin (HR 1.73, 95% CI, 1.02-2.97) had a detrimental effect. In subgroup analysis of diabetic subjects, a nonsignificant improvement in OS was observed in the metformin treated patients compared to those on other hypoglycemic agents (HR, 0.92, 95% CI, 0.55-1.55). Neither MetS nor antihypertensive drugs had any apparent effect on OS.

**Conclusions:**

Our result suggests that, among CRC patients with MetS, taking metformin and statins may improve overall survival, whereas being on insulin may negatively impact CRC prognosis. Further studies are warranted to determine the exact mechanism through which metformin, statins, and insulin exert their effects on CRC survival.

## 1. Introduction

Metabolic syndrome (MetS) represents a cluster of interrelated biochemical and physiological abnormalities with cardiovascular consequences. Different definitions of what constitutes MetS have been proposed by several institutions [1–4], and they all agree on the same basic components, namely, (1) central obesity: body mass index (BMI) of 25 kg/m^2^ or greater; (2) hypertension: antihypertensive drug administration and/or systolic blood pressure of 140 mm Hg or greater or diastolic blood pressure of 90 mm Hg or greater; (3) abnormal blood lipid levels: high triglyceride (TG) and/or low high density lipoproteins (HDLs) (i.e., TG: ≥1.7 mmol/L and/or HDL <.9 mmol/L for males; <1.0 mmol/L for females); and (4) high blood glucose level: fasting plasma glucose (FPG) of 6.1 mmol/L or greater or 2-hour postprandial plasma glucose (PPG) of 7.8 mmol/L or greater. Consequently, the pharmacological management of MetS is aimed at keeping these individual component values within acceptable limits.

Besides the cardiovascular consequences of MetS, findings from some epidemiological studies [5] and meta-analyses [6] suggest there is a link between MetS and colorectal cancer (CRC) risk and mortality though results have been inconsistent [5–8]. Other studies have equally evaluated the association between MetS management and CRC survival with mixed results [9, 10]. CRC is the most frequent cancer among Omani men and the third most common in Omani women with incidence rates of 10.2 and 8.5 per 100,000 cases for men and women, respectively [11]. In 2013, CRC accounted for 9.0% of all-cause mortality in adult Omani males and 8.3% in females [11]. Moreover, a recent observational study indicated a rising trend in type 2 diabetes, obesity, and other markers of MetS among Omani adults [12]. Given that research findings may differ by race or ethnicity [13] and the fact that effect of MetS and its treatment on CRC survival has not been previously studied in Arab population, our retrospective study was aimed at evaluating the effect of MetS on CRC survival in the Omani Arab population. We also examined if insulin, metformin, statins, and ACEI/ARBs, agents commonly employed in the treatment of MetS components, had an influence on CRC survival.

## 2. Materials and Methods

We collected data from the hospital records of 301 colorectal cancer patients diagnosed and treated in Sultan Qaboos University Hospital (SQUH) from 2006 to 2014. Patients with familial adenomatous polyposis syndrome (FAP) and those with type 1 diabetes were excluded. Sample size calculation done with the Power and Sample Size Calculation software (PS Power), version 3.1 indicated that, based on an accrual interval of 8-time units, additional follow-up of 2-time units after the accrual interval, and a hazard ratio of 1.5 in CRC subjects with MetS relative to those without MetS [14], a sample size of 206 was needed to be able to achieve 80% power in this study. However, the entire eligible CRC cases available in the dataset within the study period, 2006-2014, were 301, and this served as the sample. Demographic and clinicopathological information including age, gender, date of diagnosis, family history, body weight and height at diagnosis, presence of hypertension, diabetes, dyslipidemia, stage and grade of disease and tumor location, and type of cancer treatment were extracted from patients' medical records. Information on smoking habits and alcohol consumption was also captured and we reviewed the medication profiles of each patient and extracted information on medications for diabetes, hypertension, and dyslipidemia. Ethical approval was granted by the Sultan Qaboos University Medical Research and Ethics Committee (MREC # 1232).

### 2.1. Variables of Interest

Our outcome variable was overall survival (OS) calculated from the date of diagnosis to the date of death, censoring, or end of study on 31^st^ December 2016, whichever came first. Our main exposure variable was metabolic syndrome defined according to the American Heart Association/National Heart, Lung, and Blood Institute (AHA/NHLBI) criteria [3]. According to the AHA/NHLBI criteria, a measure of any 3 of 5 of the following conditions constitutes a diagnosis of metabolic syndrome: elevated waist circumference ≥102 cm (≥40 inches) in men and 88 cm (≥35 inches) in women, elevated triglycerides ≥150 mg/dL (1.7 mmol/L) or on drug treatment for elevated triglycerides, reduced HDL-C <40 mg/dL (1.03 mmol/L) in men and <50 mg/dL (1.3 mmol/L) in women or on treatment for reduced HDL-C‡, elevated blood pressure ≥130 mm Hg systolic blood pressure or ≥85 mm Hg diastolic blood pressure or on antihypertensive drug treatment in a patient with a history of hypertension, and elevated fasting glucose ≥100 mg/dL or on drug treatment for elevated glucose. Secondary exposure variables were medications commonly used in managing individual components of MetS: insulin, metformin, statins, and ACEI/ARBs. Each patient's medication profile was reviewed for a documentation of the medications of interest. In addition to these medications, the use of other antihypertensive medications or antidiabetic or lipid lowering agents was also captured.

We calculated body mass index (BMI) for each patient by dividing weight in kilograms by the square of height in meters and categorized it into three levels: 18.5-24.99 kg/m^2^, 25-29.99 kg/m^2^, and ≥30 kg/m^2^ according to the World Health Organization criteria [15]. BMI was further collapsed into two categories, namely, “BMI < 30 kg/m^2^” (no obesity) and “BMI ≥30 kg/m^2^” (obesity). Diabetes (yes/no), defined as fasting plasma glucose ≥ 7.0mmol/l (126mg/dl) or 2 h plasma glucose ≥ 11.1mmol/l (200mg/dl), according to the American Diabetes Association [16] cut points, was captured by reviewing the patients' admission notes for a diagnosis of diabetes or by a review of patients' drug charts for the presence of oral antidiabetic drugs or insulin. Similarly, presence of hypertension and dyslipidemia (Yes/No) were assessed from physicians' admission notes and patients' drug charts for the presence of antihypertensive drugs and statins. Age at the time of diagnosis was captured as a continuous variable. However, age was recoded into a categorical variable, “age group”, with 3 levels: ≤ 40, 41-60, and ≥ 61. Cancer stage was categorized into three according to the TNM staging system of the American Joint Committee on Cancer (AJCC ) [17] as stages 1 and 2, stage 3, and stage 4 representing localized disease that had spread to regional lymph nodes or metastasized to distant organs, respectively. Cancer treatment was categorized into 4, surgery only, surgery +systemic therapy, systemic therapy alone, and palliation, for those whose systemic intervention did not include chemotherapy, but was only aimed at controlling other symptoms of advanced disease. Smoking habits” (ever or current users) and alcohol consumption (ever or current users) were categorized as “yes or “no”.

### 2.2. Statistical Analysis

To evaluate the statistical significance of differences among proportions of categorical data, Chi-square analyses were used. The nonparametric Fisher's exact test (two-tailed) replaced the Chi-square test in cases where the expected frequency was less than 5 in any of the cells in the 2 x 2 tables. Descriptive statistics including frequencies and percentages were computed for all variables. After confirming that the proportional hazards assumption was met, we generated Kaplan-Meir survival curves separately for MetS, MetS treatment, and hypoglycemic agents and compared them with the log-rank tests. For inferential statistics, we used Cox proportional hazards models to assess the relationship between MetS and OS and between each medication (insulin, metformin, statins and ACEI/ARBs) and OS. We further performed stratified analysis in the diabetic subgroup and compared OS in diabetic subjects who took metformin versus those who were on other hypoglycemic agents. For the purpose of the subgroup analysis, “other hypoglycemic agents” included all patients on insulin and/or oral hypoglycemic agents, except metformin. The multivariable Cox proportional models were adjusted for age, cancer stage, cancer treatment, BMI, smoking, alcohol intake, and tumor differentiation. One set of multivariable model included only MetS (without the medications) and the other set included the medications (without MetS). Potential interactions among the variables of interest were tested and hazard ratios with their corresponding 95% confidence intervals were derived. Statistical significance was set at a 2-sided p value of ≤ 0.05. All analyses were conducted with the International Business Machine (IBM) SPSS Statistics version 21.0 for Windows (SPSS Inc., and Chicago, Illinois, USA).

## 3. Results

### 3.1. Patient Characteristics

Our sample consisted of a total of 301 CRC patients of which 175 (58.1%) were males.  Table 1 shows the distribution of patients' demographic, tumor characteristics, comorbidities, and medication use. The mean age of the patients was 55 ±15.15. Majority of the patients had AJCC stage 3 or 4 disease, with moderately differentiated tumor characteristics. Nearly half of the patients were hypertensive (48.5%), a third of them had elevated blood glucose (111 (36.9%)), one-fourth of patients had dyslipidemia (77 (25.6%)), and MetS were 76 (25.2%). Most of the patients received either surgery plus chemotherapy (60.1%) or chemotherapy alone (22.9%). In terms of medications for MetS management, 20.3% of patients were on insulin, 23.9% were on metformin, and 13.0% were on other antidiabetic treatments. Twenty-five point six percent of patients took statins, 17.9% were on either angiotensin converting enzyme inhibitors (ACEI) or angiotensin receptor blocker (ARB), and 31.8% were on other types of antihypertensive agents.

### 3.2. Survival Outcomes

**Figure 1:**
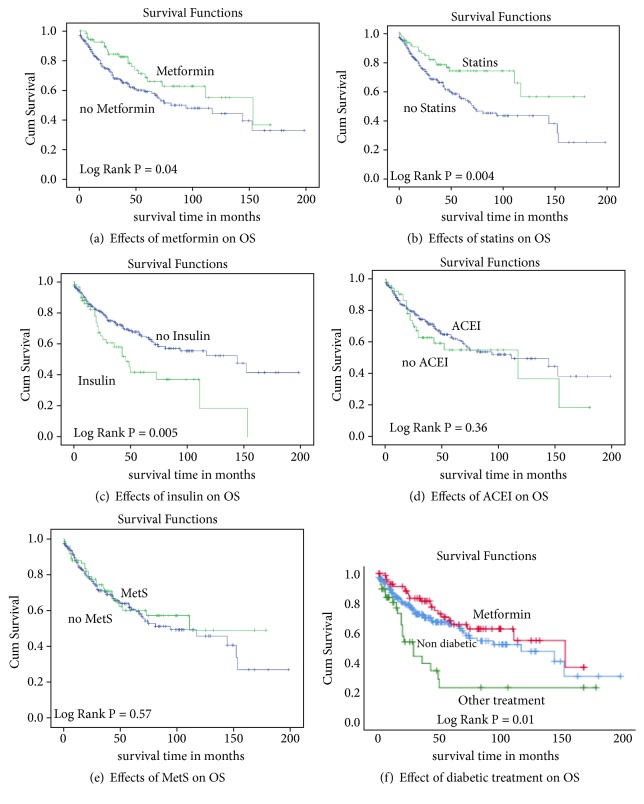


Table 2 and Figure 1 show the median OS in months, the hazard ratios (HR), adjusted for potential confounding variables, and corresponding 95% confidence intervals (CI) for MetS and MetS treatment. Being on metformin conferred a significantly longer median OS compared to not taking metformin at 49.9 months versus 19.92 months, respectively (HR, 0.46; 95% CI, 0.25-0.84) (Table 2, Figure 1(a)). Similarly, those taking statins had significantly longer median OS at 111 months compared to 72 months for those who did not (HR, 0.58; 95% CI, 0.30-0.97) (Table 2, Figure 1(b)). On the other hand, those on insulin had a significantly lower median OS at 46 months compared to 144 months for those not on insulin (HR, 1.73; 95% CI, 1.02-2.97) (Table 2, Figure 1(c)). However, MetS had no statistically significant effect on OS in univariate and multivariate analysis. Median OS for patients with MetS was 114 months versus 94 months for those with no MetS. The adjusted HR was 1.01 and 95% CI, 0.64-1.59 (Table 2, Figure 1(e)). Likewise, ACEI/ARB (HR, 1.46 (95% CI, 0.89-2.38) had no effect on OS in univariate and multivariate analysis (Table 2, Figure 1(d)). In the diabetic subgroup stratified by metformin use, there was a statistically significant difference in overall survival between the three groups (log-rank P = 0.001). Median OS in months was more favorable in the metformin group at 153 months (55.5-250.5) compared to 29 months (16.12-41.84) in subjects who were on other antidiabetic agents and 117 months (59.29-174.71) in nondiabetic subjects (Table 3, Figure 1(f)). However, in the univariate and multivariable adjusted Cox proportional regression, the effect of metformin exposure on OS (HR, 0.92; 95% CI, 0.55-1.55) was not statistically significant (Table 3).

## 4. Discussion

To the best of our knowledge, this is the first study to examine the effects of MetS and its treatment on overall survival (OS) in CRC patients in Oman. In this study, we found that use of metformin was associated with favorable but nonstatistically significant survival outcomes. The use statins also had a protective association with OS. However, insulin use was associated with unfavorable survival outcomes. There was no association between MetS, use of ACEI/ARBs or other antihypertensives, and CRC survival.

Consistent with findings from recent studies [18, 19], we found that use of metformin had a protective effect on OS. However, unlike these previous studies, the subgroup analysis of diabetic subjects in our study revealed that though the use of metformin was associated with favorable survival (Table 3), it failed to reach statistical significance. It should be noted that the diabetic subjects in our study were only 111 (Table 1) and may not have provided enough power for the subgroup analysis. This lack of sufficient power could have resulted in the observed inconsistency with previous results. Our findings should therefore be interpreted with caution until additional studies with larger samples have been done. Another interesting finding from our study was that, in the subgroup analysis (Table 3), subjects who took metformin had a longer OS (153 months) compared to the nondiabetic group (117 months), suggesting that, irrespective of the presence of comorbid diabetes, metformin might confer survival advantage for CRC patients in general. Metformin is a biguanide used to treat hyperglycemia in most diabetic patients and has been reported to have a protective effect on breast and colon cancers [20]. Plausible mechanisms by which metformin exert its anticancer effects have been advanced by some studies [21–23]. One of such studies stated that metformin inhibits the mammalian target of rapamycin complex 1 (mTORC1) pathway [21]. The mTORC1 pathway plays a key role in metabolism, growth, and proliferation of cancer cells [22]. Moreover, metformin was found to reduce insulin level, inhibit insulin/IGF signaling pathways, and modify cellular metabolism of insulin in normal and cancer cells [23]. Increased tissue availability of circulating insulin/IGF1 and upregulation of insulin/IGF receptor signaling pathways has been implicated in the formation of different cancers in observational studies [24, 25]. The potential benefits of metformin are currently the focus of many studies on aging, autoimmune disorders, tuberculosis, erectile dysfunction, and cancers [26]. The use of metformin as a possible preventive intervention for CRC is one of such studies [27].

In our study, use of statins improved OS similar to the findings from other studies [28–30]. In a retrospective study that investigated the effects of statins on overall survival of patients with a diagnosis of colon cancer in the Netherlands, statin use was associated with a 34% reduction in colorectal cancer death in the subset of patients with bone morphogenetic protein (BMP) signaling irrespective of their KRAS status [28]. Similar results were obtained from a large population-based cohort in the United Kingdom [29], which showed that statin use after diagnosis of colorectal cancer was associated with longer rates of survival (HR, 0.71; 95% CI, 0.61 - 0.84). A Scottish CRC cohort study and updated meta-analysis showed that statin use was associated with improved survival. However, the strength of the associations varied markedly between studies [30]. In one study, however, statin use was not associated with improved survival either independently or when stratified by p53 and 3-hydroxy-3-methylglutaryl coenzyme-A reductase (HMGCR) expression [31]. Given these inconsistencies, the pathophysiological pathway through which statins influence CRC survival warrants further research.

In our study, use of insulin was associated with worse OS. Consistent with our result, detrimental effect of insulin on CRC survival has been demonstrated in some studies involving diabetic patients treated with insulin [32–34]. In one such study [32], the authors reported a more than 50% higher risk of CRC mortality in diabetic patients on insulin compared to noninsulin users. In another study [33], an overall excess CRC mortality in female diabetic patients treated with insulin compared to noninsulin treated participants was observed. In a more recent study, increased levels of insulin correlated with worse overall survival in patients with nonmetastatic CRC [34]. According to some authors [35], there are several potential explanations for the relationship between insulin and increased CRC mortality. For example, hyperinsulinemia from impaired glucose metabolism and insulin resistance may contribute to increased tumor growth [20]. Secondly, cancer patients with diabetes might be treated less aggressively than those without diabetes [35]. It could also be due to poorer response to cancer treatment that increased infection risk or intraoperative mortality in diabetic cancer patients relative to nondiabetics [36]. Direct stimulation of cell proliferation and downstream activation of mitogen-activated protein kinase (MAPK) by insulin has also been implicated [37].

Study results on the effects of MetS and its treatment on colorectal cancer survival have generally been inconsistent [5, 14]. In one large retrospective cohort study, the authors examined data to determine the effects of MetS and its components on overall as well as recurrence-free survival among 36,079 colon cancer patients. Their result showed that MetS had no apparent effect on overall as well as recurrent-free survival [38]. In another large study, a cluster of MetS components increased CRC mortality significantly in both males and females [39]. It has been suggested that the conflicting results on the effects of MetS and CRC mortality reported by observational studies may be due to different definitions of MetS in the studies and failure to control for potential confounding variables such as disease stage, cancer type, and type of treatment [14]. In our study, we did not examine the effects of MetS separately for colon and rectal cancer; therefore, we were unable to determine how this would have affected our results.

Our result showed that use of ACEI or ARB had no effect on CRC survival contrary to findings from other studies [40, 41]. Angiotensin I-converting enzyme inhibitors (ACEIs) and angiotensin II receptor blockers (ARBs) are widely used antihypertensive drugs. They regulate arterial blood pressure via the renin-angiotensin system (RAS). The RAS induces angiogenesis and tumor proliferation by promoting vascular endothelial growth factor (VEGF) or epidermal growth factor receptor (EGFR) expression [42, 43]. Moreover, angiotensin II has been shown to stimulate tumor growth [44]. ACEIs suppress the local RAS by reducing the production of angiotensin II, whereas ARBs selectively block the action of angiotensin II type 1 receptor (AT1R). Studies have reported reduced rates of distant metastasis and decreased mortality risk in ACEI or ARB users with lung and colorectal cancers [40, 45]. Inherent differences, such as ethnicity, between our patient population and that of previous studies may, in part, account for our discrepant findings.

Several limitations may have affected our study results. First, our study was retrospective in design so we were limited to data available in patients' hospital records which may not have been consistent or complete. Information on predictor variables was captured as snapshot of patients' hospital records and did not take into account any changes that may have occurred as patients' disease progressed. For example, BMI was calculated from patients' height and weight at the time of diagnosis. Cancer patients experience weight changes with progression of their disease; therefore any decreases in weight among the patients would have affected BMI and, invariably, affect our result.

BMI was used as a proxy for central obesity because we could not measure waist circumference given the retrospective nature of our study. Waist circumference is a more accurate indicator of central obesity; therefore, prospective studies that can measure patients' waist circumference are warranted. Specific cancer treatments such as chemotherapy as well as the medications used to manage the components of MetS and other comorbid conditions may have also altered tumor behaviour and affected survival. For example, we could not determine if there were any changes in diabetic treatment during the follow-up period and how this might have affected our result. Additionally, in the subgroup analysis, “other diabetes treatments” included not only patients who were on other oral agents, but also those on insulin. Given that use of insulin might represent the more advanced stages of diabetes, this may have contributed, in part, to the poorer OS observed in this group compared to the metformin group.

Lastly, cancer is a progressively terminal disease with relatively worse prognosis for patients in later stages of disease. In our study, more than two-thirds of our total study subjects were of AJCC stage 3 or 4 disease; therefore, cancer progression might have affected OS. This study was a single center study, carried out with records of CRC patients from one of the two hospitals that treat cancers in Oman; therefore, the findings from this research may not be representative of the CRC population in Oman and should be interpreted with caution.

## 5. Conclusion

Our findings suggest that among Omani CRC patients with MetS, metformin and statins might lower the risk of dying. On the other hand, insulin appeared to be detrimental to overall survival. With the current trend towards personalized cancer treatment, prospective studies are needed to better understand the exact mechanism by which metformin, statins, and insulin exert their effect on CRC survival.

## Figures and Tables

**Table 1:** Demographic and clinicopathological characteristics of the sample N = 301.

*Variable*	Number	Percentage (%)
*Age (y)*		
*Mean ± SD (y)*	55	±15.15
*≤40*	45	15
*41-60*	131	43.4
*≥61*	125	41.5
*Gender*		
Male	175	58.1
Female	126	41.9
*AJCC stage*		
1&2	78	25.9
3	104	34.6
4	119	35.9
*Tumor differentiation*		
Well	36	12.0
Moderate	209	69.4
Poor	46	15.3
*Cancer Treatment*		
Surgery only	34	11.3
Surgery +Systemic therapy	181	60.1
Systemic therapy only	69	22.9
No treatment	17	5.6
*Obesity*		
No	207	68.8
Yes	95	31.2
*Elevated glucose/DM*		
No	190	63.1
Yes	111	36.9
*Hypertension*		
No	155	51.5
Yes	146	48.5
*Dyslipidemia*		
No	224	74.4
Yes	77	25.6
*MetS*		
No	225	74.8
Yes	76	25.2
*Insulin use*		
No	240	79.7
Yes	61	20.3
*Metformin use*		
No	229	76.1
Yes	72	23.9
*Statin use*		
No	224	74.4
Yes	77	25.6
*ACEI/ARB use*		
No	247	82.1
Yes	54	17.9
*Other antiHTN*		
No	206	68.4
Yes	95	31.8
*Aspirin Use*		
No	248	82.4
Yes	53	17.6
*Anti-diabetic treatment*		
No Diabetes	190	63.1
Metformin	72	23.9
Other treatment	39	13.0

*Status*		
Died	105	34.9
Censored	196	61.5

Note: AJCC: American Joint Committee on Cancer, ACEI: angiotensin converting enzyme inhibitors, DM: diabetes mellitus, antiHTN: antihypertensive, MetS: metabolic syndrome, SD: standard deviation, and other treatments: insulin and/or other oral hypoglycemic agents, except metformin.

**Table 2:** Effect of metabolic syndrome and treatment on CRC survival.

Variable	Crude HR	95% CI	*∗*Adjusted HR	95% CI
*MetS*				
No	Ref	-		-
Yes	0.89	0.57-1.38	1.01	0.64-1.59
*ACEI use*				
No	Ref	-		-
Yes	2.06	1.19-3.56	1.46	0.89-2.38
*Other anti-HTN*				
No	Ref	-		-
Yes	1.89	0.24-14.67	1.74	0.21-14.70
*Metformin use*				
No	Ref	-		-
Yes	0.38	0.20 -0.71	0.46	0.25-0.84
*Insulin use*				
No	Ref	-		-
Yes	1.28	1.09 -1.75	1.73	1.02-2.97
*Statin use*				
No	Ref	-		-
Yes	0.42	0.25-0.74	0.58	0.30-0.97

MetS: metabolic syndrome. *∗*Adjusted for age, sex, and cancer stage and tumor differentiation, cancer treatment, BMI, alcohol, and smoking.

**Table 3:** Effect of type of antidiabetic treatment on CRC survival.

Variable	OS (months)	Crude HR	95% CI	*∗*Adjusted HR	95% CI
Non Diabetic	119	Ref	-	Ref	-
On metformin	153	0.74	0.46-1.20		
Other treatment*∗∗*	29	2.18	1.31-3.63	2.11	1.19-3.75

*∗*Adjusted for age, sex, and cancer stage and tumor differentiation, cancer treatment, BMI, alcohol, and smoking, *∗∗* other treatment = insulin and/or other oral hypoglycemic agents, except metformin, and OS = overall survival.

## Data Availability

The data used to support the findings of this study are restricted by the Medical Research and Ethics Committee of Sultan Qaboos University in order to protect patient privacy.
